# Stress Coping Strategies in the Heart: An Integrated View

**DOI:** 10.3389/fcvm.2018.00168

**Published:** 2018-11-21

**Authors:** Marek Michalak, Luis B. Agellon

**Affiliations:** ^1^Department of Biochemistry, University of Alberta, Edmonton, AB, Canada; ^2^School of Human Nutrition, McGill University, Ste. Anne de Bellevue, QC, Canada

**Keywords:** endoplasmic reticulum stress, proteostasis, cardiac cells, stress responses, membrane contact sites

## Abstract

The heart is made up of an ordered amalgam of cardiac cell types that work together to coordinate four major processes, namely energy production, electrical conductance, mechanical work, and tissue remodeling. Over the last decade, a large body of information has been amassed regarding how different cardiac cell types respond to cellular stress that affect the functionality of their elaborate intracellular membrane networks, the cellular reticular network. In the context of the heart, the manifestations of stress coping strategies likely differ depending on the coping strategy outcomes of the different cardiac cell types, and thus may underlie the development of distinct cardiac disorders. It is not clear whether all cardiac cell types have similar sensitivity to cellular stress, how specific coping response strategies modify their unique roles, and how their metabolic status is communicated to other cells within the heart. Here we discuss our understanding of the roles of specialized cardiac cells that together make the heart function as an organ with the ability to pump blood continuously and follow a regular rhythm.

## Introduction

**Figure 1:**
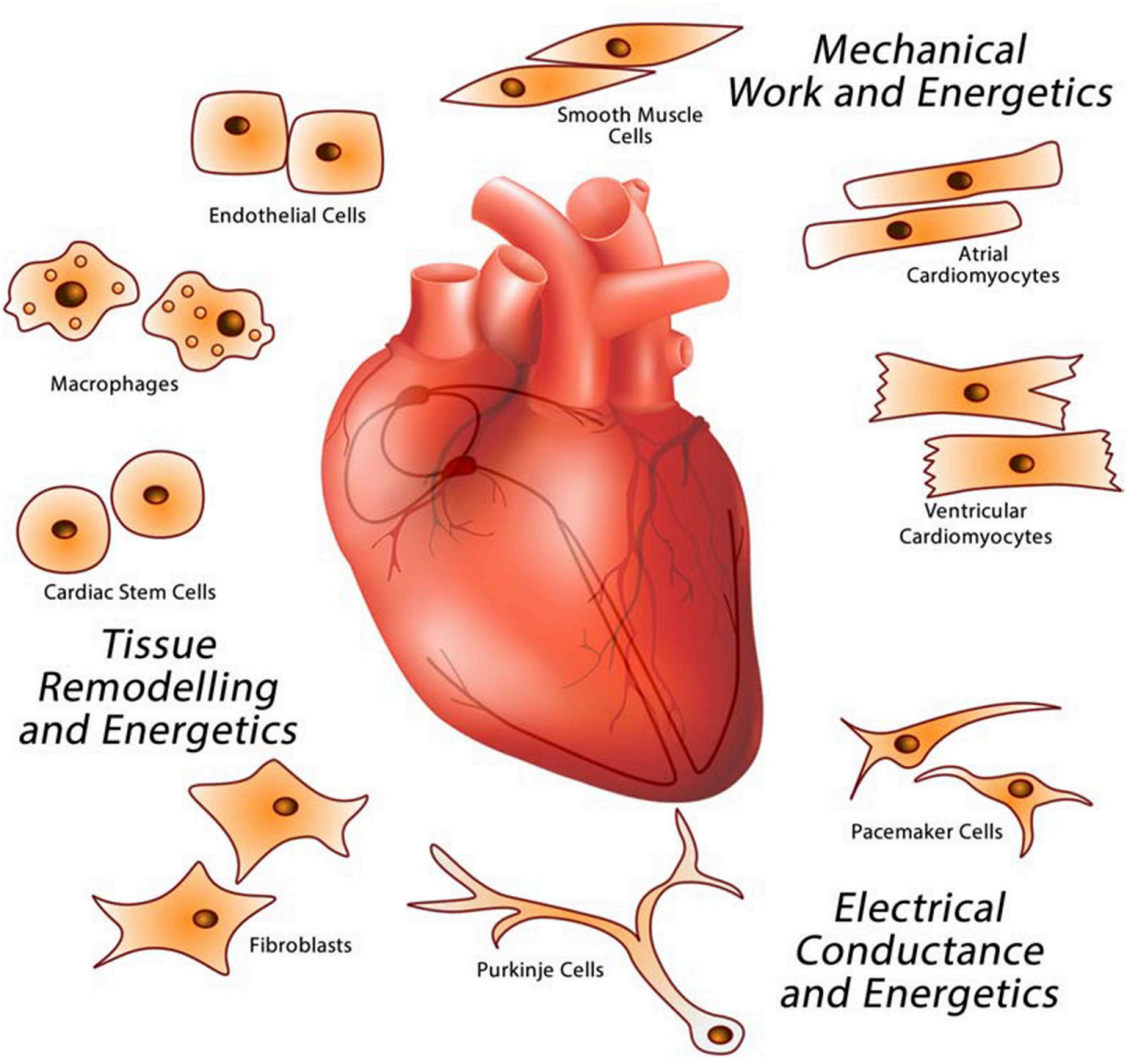
Major cardiac cell types that make up the heart. The heart is a multicellular organ comprised of different cell types responsible for providing specific functions related to electrical conductance (macrophages, pacemaker, and Purkinje cells), mechanical work (cardiomyocytes), and tissue remodeling (endothelial cells, fibroblasts, macrophages, and stem cells).

Non-communicable diseases, in particular cardiovascular diseases and diabetes, now account for over half of all mortalities in the world (1). Acquired heart diseases have devastating consequences and are the most common cause of adult death. Environmental factors, lifestyle choices, and gene variations collectively contribute to the dysfunction of cardiac cells and development of a variety of heart diseases which when left untreated lead to diminished pump function, heart failure, and eventual death (2). There are many specific risk factors for heart diseases, and recent reports indicate that many diseases of the heart are triggered by and/or associated with stress and cellular stress coping response mechanisms (3). For example, the energy dense diets commonly consumed in industrialized societies, and those undergoing nutrition transition, are associated with cardiovascular diseases (1, 4–7). Myocarditis, a disease caused by viral infection, is characterized by inflammation and disruption of the electrical pathways that support normal sinus rhythm which triggers heart failure (8). The ability of cardiac cells to respond to cellular stress is important for the maintenance of cardiac health (3). To carry out its primary purpose as an organ, the heart takes advantage of the functions of specialized cell types to manage energetics, electrical conduction, mechanical work and tissue remodeling (Box 1, Figure 1). The aim of this review is to discuss the coordination of the various activities occurring simultaneously in the different cell types that make up the heart and enable it to continuously pump blood within the body.

Box 1 **The Heart.**

The heart is a complex biological structure that contracts rhythmically and continuously to distribute blood to the lungs and the rest of the body. It is comprised of unique cell types with dedicated functions that interact together forming a complex networked organ (Figure 1). The human heartbeats an average 80 times a minute to circulate up to 8,000 l of blood a day. Each cycle of the continuous cardiac sinus rhythm is comprised of a systole and a diastole and depends on the cardiac action potential transmitted as electrical impulses generated by the cardiac conductive system (SA, sinoatrial node and AV, atrioventricular node) that functions independently of the central nervous system. Cardiac cells also have endocrine functions releasing natriuretic peptides and cytokines to influence the blood pumping function of the heart. Of the many cell types found in the heart, atrial and ventricular cardiomyocytes form the heart myocardium. Cardiomyocytes occupy the majority of the volume of the mammalian heart (9). Of all nonmyocytes in the healthy adult heart, over 60% are endothelial cells, 5% are hematopoietic-derived cells and 20% are fibroblasts (10), with the remainder being made up of Purkinje and pacemaker cells, smooth muscle cells, and stem cells. Endothelial cells form the endocardium, the interior lining of cardiac valves, whereas smooth muscle cells contribute to coronary arteries and inflow and outflow vasculature. The pericardium is enriched in fibroblasts and connective tissue and allows the heart to function in a frictionless environment. Cardiac stem cells play a role in cardiac repair (11). Pacemaker cells and cells that make up Purkinje fibers in the electrical conduction system are specialized cardiomyocytes that produce and conduct electrical impulses. Cardiac macrophages are interspersed among other cardiac cell types (12–14).

**Cardiomyocytes:** Cardiomyocytes contain highly organized sarcomeres that facilitate contraction to fulfill the mechanical work of the heart (15). Neonatal and adult cardiomyocytes, HL-1 cardiomyocytes, ANF-T-antigen immortalized cardiomyocytes, and H2C9 myoblasts have been used in culture to study cardiomyocyte function (15). Pluripotent embryonic stem cells and induced pluripotent stem (iPS) cells have also been used to generate cardiomyocytes (16), and these cells contract and relax spontaneously in culture. Cardiac hypertrophy is primarily the result of cardiac myocyte enlargement and this feature can be experimentally recapitulated in tissue culture. Study of these models have yielded detailed knowledge about signaling pathways that control cardiomyocyte activity and have served as platforms for screening of pharmacological agents targeting cardiomyocyte-specific functions related to energetics, contractile and electrical functions. Atrial and ventricular cardiomyocytes do not have identical gene expression programs or Ca^2+^ handling (17–19). For example, atrial cardiomyocytes, unlike ventricular cardiomyocytes, possess endocrine function to influence blood pressure, renal function and ion balance (20, 21).

**Pacemaker Cells:** Cardiac pacemaker cells control the heart rate. These cells are concentrated in the SA node and are responsible for generating and propagating an action potential to the AV node to coordinate the mechanical action of cardiomyocytes. Pacemaker cells are sensitive to β-adrenergic stimulation and also might be controlled by micropeptides that affect their Ca^2+^ handling (22, 23). Dysfunction of pacemaker cells causes cardiac arrhythmias and heart block, which have been linked to heart failure.

**Purkinje Cells:** These cells form cardiac Purkinje fibers which specialize in rapid transmission of electric impulses in the heart. These cells predominantly express connexin40 and their action potential is longer than that of ventricular cardiomyocytes. Many ventricular arrhythmias, including long QT syndrome, are initiated in the Purkinje fiber conduction system (24).

**Fibroblasts:** Cardiac fibroblasts are highly responsive to cardiac injury and produce components of the extracellular matrix (ECM) such as periostin, fibronectin, collagen type I, III, V, VI (25, 26). Cardiac stem cells and endothelial cells may also contribute to ECM assembly (27). Cardiac fibroblasts also participate in matrix degradation, conduction system insulation, cardiomyocytes electrical coupling, vascular maintenance, and hemodynamic stress sensing (28, 29). These cells mediate the formation of fibrotic scars in response to cardiac injury (27).

**Endothelial Cells:** Cardiac endothelial cells are found in the aorta, coronary artery, pulmonary artery, and microvasculature. They play a role in regulating and maintaining the endocardium, and in the myocardial capillaries where endothelial cells directly interact with adjacent cardiomyocytes (30). Cardiac endothelial cells, like vascular endothelial cells, produce and release a variety of autocrine and paracrine agents, such as nitric oxide (NO), endothelin, prostaglandin I, and angiotensin II, which directly influence cardiac metabolism, growth, contractile performance, and rhythmicity (30). Endothelial dysfunction due to increased production of reactive oxygen species (ROS) and reduced synthesis of NO are key features of myocardial ischemia/reperfusion injury (31).

**Pericytes:** Pericytes control important physiological processes such as angiogenesis, blood flow and vascular permeability (32, 33). In the heart, these cells play a role in cardiac regeneration. Dysfunction of cardiac pericytes contributes to cardiovascular diseases including hypertension, myocardial edema, and post-ischemic coronary no-reflow.

**Smooth Muscle Cells:** Coronary artery smooth muscle cells are an integral part of cardiac vasculature and contribute to inflow and outflow vasculature. Coronary vasculature supplies the heart with nutrient-containing blood. Atherosclerosis or thrombosis can cause acute disruption of vascular function leading to myocardial infarction (34). As atherosclerosis worsens, the presence of LDL and atherogenic cytokines stimulate vascular smooth muscle cells to alter extracellular matrix composition and thus cause pathogenic vascular remodeling (35).

**Macrophages:** Macrophages are typically viewed as mobile and appear at sites of injury in response to stress and inflammation (36). However, certain populations of macrophages that are found in some organs, such as in the heart, are produced during organogenesis (36, 37). Resident cardiac macrophages are interspersed between cardiac cells (12, 14, 36) and have recently been implicated in the cardiac electric conduction system (37). Macrophages appear to be integral components of the AV node, connecting the atrial cardiomyocytes with ventricular cardiomyocytes via connexin43-containing gap junctions (37). Dysfunction of AV macrophages or inability of macrophages to communicate with cardiomyocytes induces AV block (37). Blood monocyte-derived and cardiac resident macrophages also contribute to inflammatory and remodeling stages of cardiac disease by phagocytosis of necrotic and apoptotic cells, release of proteases, pro-inflammatory cytokines/chemokines and ROS, and promotion of collagen production (38). Each of the different cell types that make up the heart fulfill unique roles to promote the function of the heart as an organ. Aberration of specific functions provided by each of the specialized cardiac cell types likely manifest in different ways and explains the heterogeneity of cardiac diseases. In acquired cardiac disorders, the insults that perturb cellular functions originate from the environment. The questions that arise relate to the relative sensitivity of the different cardiac cell types to specific stressors, the manner in which the different cell types respond to these stressors, and whether the status of affected cells is communicated to other cell types that make up the organ.

## Cellular stress coping strategies in the heart

**Figure 2:**
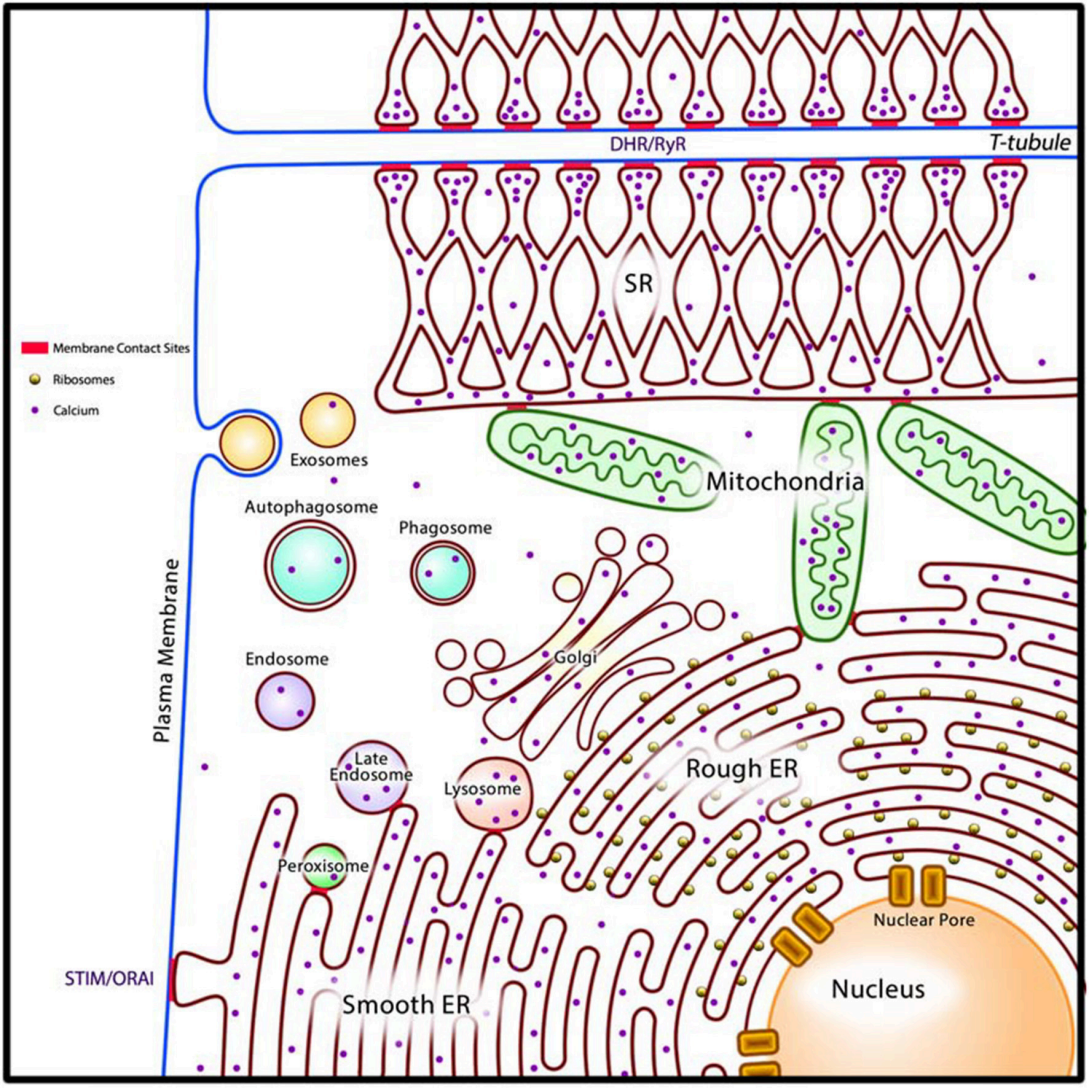
A generalized model of the cardiac cellular reticular network (CRN). Intracellular organelles (rough and smooth ER, SR in cardiomyocytes and smooth muscle cells, Golgi apparatus, lysosomes, membranous components of the endocytic pathway, peroxisomes, and nuclear envelope) are organized into the cellular reticular network (CRN). The CRN is linked by membrane contact sites (MCS, in red) allowing for rapid exchange of molecules between component organelles. In cardiomyocytes the MCS that join SR and T-tubules (DHR/RyR) allow communication between the plasma membrane and the SR to drive E-C coupling. Smooth ER form MCS with the plasma membrane (STIM/ORAI) to drive store-operated Ca^2+^ entry (SOCE). DHR, plasma membrane dihydropyridine Ca^2+^ channel receptor; RyR, SR ryanodine receptor/Ca^2+^ channel; STIM, ER Ca^2+^ sensor; ORAI, plasma membrane Ca^2+^ channel; ER, endoplasmic reticulum.

External factors are important risk factors for promoting the development of acquired cardiac disorders. For example, in viral infection, viruses hijack cellular processes and divert cellular resources toward viral replication. Xenobiotics that directly damage or disrupt protein function have detrimental impacts on the integrity of the cellular machinery. Similarly, deficiency of certain types of nutrients such as minerals and vitamins or excess nutrients that serve as fuel, negatively affect cellular metabolism since many of these compounds serve as enzyme co-factors as well as regulate gene expression (7). Lack of oxygen, as in ischemia, compromises the ability of cells to carry out oxidative phosphorylation. Long term perturbation of cellular energetics subsequently leads to loss of proteostasis (unfolded proteins, damaged proteins) and lipidostasis (damaged lipids, disordered membranes) within the cellular reticular network (CRN) (Box 2, Figure 2) (39). These situations have detrimental consequences to the heart as the organ requires a continuous supply of ATP to maintain its function.

Box 2 **The cellular reticular network (CRN).**

All cardiac cells, as with other eukaryotic cells, consist of intracellular organelles namely the ER, Golgi apparatus, lysosomes, membranous components of the endocytic pathway, peroxisomes, and nuclear envelope (luminally connected to the ER) (Figure 2). We propose that these organelles are organized into the cellular reticular network (CRN) (3, 39), a linked intracellular network of specialized membrane systems, which work in concert to facilitate cell function. Organelles coordinate their activities to maintain homeostasis and fulfill their cellular roles under a variety of metabolic conditions. For example, the ER is responsible for many key cellular processes including the synthesis, folding, posttranslational modification, and transport of proteins; synthesis of lipids and sterols; assembly and trafficking of membranes; drug and xenobiotic detoxification; storage and release of intracellular Ca^2+^; regulation of gene expression, cellular energetics; and signaling to the nucleus, cytoplasm, mitochondria, and plasma membrane (3, 40, 41). Nearly all these processes utilize energy and thus rely on mitochondria for ATP production. Synthesis of membrane-bound and exported proteins involves the Golgi apparatus. Moreover, synthesis of cellular lipids involves the activities of enzymes resident in the ER, mitochondria, and peroxisomes (42). Coordination of these cellular processes is achieved in part through membrane contact sites (MCS), which are formed between closely apposed organellar membranes including the plasma membrane (Figure 2) (43–50).

The ER is the dominating component of the CRN. In some cells, the ER is further subspecialized into rough ER, such as in secretory cell types, and smooth ER, such as in cells that actively synthesize and metabolize lipids. In cardiomyocytes and smooth muscle cells, in addition to perinuclear rough and smooth ER, the ER is further subspecialized into sarcoplasmic reticulum (SR) which is instrumental in the regulation of excitation-contraction (E-C) coupling to facilitate muscle contraction and relaxation (51). There are two well defined structural and functional regions of the SR: the longitudinal SR, a membrane network around myofibrils extending into the junctional SR, a membrane network with multiple MCS with T-tubules, which are invaginations in the plasma membranes of muscle cells such as cardiomyocytes (Figure 2) (51, 52). The longitudinal SR is enriched with Ca^2+^-transport ATPase (SERCA), responsible for rapid removal of Ca^2+^ from the cytoplasm to initiate muscle relaxation (51, 52). The junctional SR contains the ryanodine receptor/Ca^2+^ channel (RyR), responsible for Ca^2+^ release to the cytoplasm to initiate muscle contraction, and calsequestrin, a major Ca^2+^ binding protein of muscle (51–53). The existence of the SR in cardiomyocytes and smooth muscle cells allows these cells to carry out cellular processes related to mechanical functions, notably E-C coupling, without compromising other important Ca^2+^ requiring cellular processes that are normally associated with rough ER and smooth ER (54).

Overall, the ER/SR remains the main driver of intracellular Ca^2+^ signaling and the maintainer of cellular Ca^2+^ homeostasis. Other components of the CRN contribute to Ca^2+^ homeostasis and signaling (55), and this likely is facilitated through MCS that link the various organelles. The release of Ca^2+^ from ER stores triggers Ca^2+^ influx from extracellular space via the store-operated Ca^2+^ entry (SOCE) mechanism (56–58). SOCE is the primary mechanism for replenishment of depleted ER Ca^2+^ stores in virtually all non-excitable cells (56–58). The main molecular components of the SOCE include the ER-associated luminal Ca^2+^ sensor STIM (stromal interacting molecule) and plasma membrane Ca^2+^ channel ORAI (56–58). In cardiomyocytes, SOCE operates in parallel with the E-C coupling mechanism to maintain basal Ca^2+^ homeostasis (59–63), but it is not yet clear if SOCE and cardiac E-C coupling mechanisms interact. In cardiomyocytes, increased Ca^2+^ influx due to SOCE prolongs the duration of the action potential, a contributing factor to the development of cardiac diseases (64).

The advantage of a well-developed CRN is the compartmentalization of different cellular processes such as E-C coupling, protein and lipid synthesis, ATP synthesis, and Ca^2+^ signaling, within optimal environments. For the CRN to work properly and have the ability to respond to cellular stress there must be a mechanism to allow efficient signal transmission between different compartments of the network. In the context of organs, such as the heart, intracellular communication within the CRN inevitably leads to intercellular communication that enables the function of the organ as the whole. Although not intensively studied in the heart, with the exception of junctional SR-T-tubules connection, MCS are important structures enabling the specialized roles of different cardiac cells. SR-T-tubules MCS play a role in initiation of the E-C coupling (51) while STIM-ORAI MCS support intracellular Ca^2+^ signaling in all cardiac cells (63). ER/SR-mitochondria MCS are necessary for energy supply of mechanical and electrical functions of the heart (65). Mitochondria-ER contacts are also the sites of mitochondrial fission (66) and are associated with mitochondrial DNA replication (67). Furthermore, MCS are also involved in the recruitment of the mitophagy apparatus for removal of defective mitochondria (68). Under ER/SR stress conditions there is a proliferation of MCS between ER/SR and mitochondria to increase the efficiency of Ca^2+^ transfer into mitochondria from the ER/SR as part of the adaptive response (69, 70).

The emerging concept is that MCS provide structures and additional mechanisms for efficient sensing, signaling and exchange of molecules among the different components of the CRN. In the heart, this offers individual cardiac cells the capability to facilitate a rapid intracellular movement of molecules both under physiological conditions and in response to stress. Understanding the formation, function and maintenance of MCS in the CRN under physiological and pathological settings will provide insight into the importance of the CRN in the elaboration of adaptive responses that are stimulated by cellular stress coping response mechanisms.

**Figure 3:**
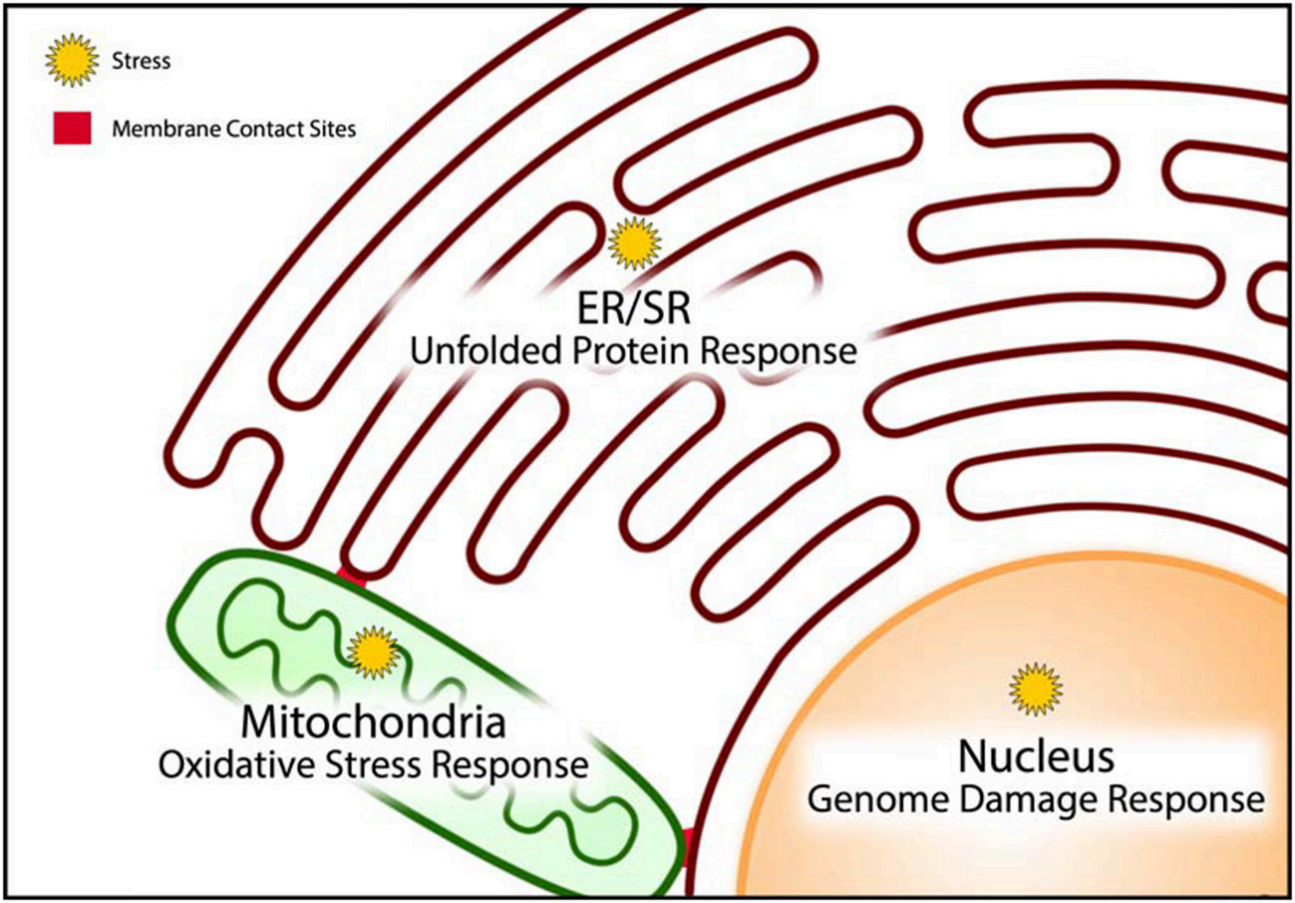
Stress coping response mechanisms. Cellular organelles have resident stress coping response mechanisms that are designed to mitigate the impact of stress and minimize damage caused by stress. The unfolded protein response, oxidative stress response, and genome damage response mechanisms operate predominantly in the endoplasmic/sarcoplasmic reticulum (ER/SR), mitochondria, and nucleus, respectively.

To mitigate cellular stress and re-establish homeostasis, cardiac cells must activate stress coping response mechanisms (3, 71, 72) (Figure 3). At the cellular level, failure to adapt to cellular stress leads to cell death. For survival, cells must successfully cope with the cellular stress, either by eliminating the source of the stress or by altering cellular metabolism to circumvent the negative effects of long term cellular stress. In the latter case, modification of cellular function to regain homeostasis without eliminating cellular stress permits cells to escape cell death but contributes detrimental effects to the organism since the adapted cells have modified function which is reflected in changes their gene expression program that can also be propagated to descendant cells through epigenetic modification of the genome (72). Indeed, epigenome remodeling of cardiac cells has been associated with aging, exposure to environmental factors and cardiac disease states (73–77).

The unfolded protein response (UPR) pathway is a well described stress coping mechanism to regain proteostasis in the ER by activation of several signaling pathways associated with ER-associated protein degradation (ERAD), autophagy, apoptosis and inflammatory signaling (3, 78–83) (Figure 3). Proteostasis is essential to all the components of the CRN, but it is especially important for the ER to maintain proteostasis because this organelle is the site of membrane protein and lipid synthesis (Box 2, Figure 2). If uncorrected, the loss of proteostasis inevitably causes loss of lipidostasis since membrane bound proteins may not be able to function properly in an environment with aberrant lipid composition (84). For example, a recent study revealed that membrane lipid composition is closely tied to the ability of SERCA to fulfill its functions in Ca^2+^ transport (85) and therefore cardiac relaxation.

It is important to note that the UPR has a high demand for energy to relieve ER stress. ATP synthesis through oxidative phosphorylation in mitochondria creates ROS. These highly reactive species are normally quenched by an elaborate and efficient scavenging system, involving a variety of endogenous antioxidant proteins (such as glutathione, thioredoxin) and transcription factors (such as Nrf2, NF-kB) (86–88), thereby preventing chemical damage to cellular proteins and DNA. ROS are also formed by metal catalyzed oxidation, various oxidases, uncoupled NO synthase and myeloperoxidase from infiltrating monocytes and neutrophils (89). Although ROS are commonly described as damaging agents, recent studies suggest that ROS may also function as signaling molecules. Increased and uncontrolled oxidation reactions within the mitochondria induce mitochondrial membrane permeability transition (MMPT) causing the leakage of mitochondrial contents, such as cytochrome C, ROS and Ca^2+^, into the cytoplasm (Figure 3). This event is indicative of unrecoverable mitochondrial damage and signals the initiation of controlled cell death by apoptosis (89–91). In doxorubicin-induced cardiac toxicity, mitochondrial localization of Bnip3, a BH3-only protein Bcl-2-like interacting protein 3, increases ROS production, and induces the loss of mitochondrial membrane potential, driving MMPT pore opening and leading to both apoptotic and necrotic cell death (92).

Under optimal conditions, mitochondria form a tight network that efficiently synthesize ATP without the accumulation of damaging ROS (93, 94) and this might be associated with longevity. The mitochondrial network has been shown to break down *functionally and morphologically* under conditions of fuel excess, and is accompanied by increased production and accumulation of ROS (95–97). At times of fuel insufficiency, autophagy may serve as a source of substrates in addition to the removal of defective cellular components. Thus, effective communication among the components of the CRN (Figure 2) and coordination of stress coping response mechanisms likely enables the cell to minimize damage to itself as well as to prevent the spread of damaging molecules to neighboring cells. The coordination of these strategies is perhaps best illustrated by the integrated induction of MMPT from the UPR to signal cell death in the case of unrecoverable damage, and by autophagy to remove damaged cellular components that can be used as temporary sources of building materials or fuel substrates (Figure 3) (98).

At present, it is not known if all cardiac cell types have similar sensitivity to inducers of cellular stress, whether the specific stress sensors and coping strategies are configured in the same way in all cardiac cell types, how specific coping response strategies modify their unique roles, and how their metabolic status is communicated to other cells within the heart.

## Cardiac energetics and stress coping responses

Cellular energetics is a basic function of all cells. Like other cells in the body, cardiac cells can use a variety of substrates to produce the energy to maintain cellular processes associated with growth and proliferation, cell signaling and metabolic homeostasis. Cardiomyocytes of the fetal heart generate ATP mainly by glycolysis whereas cardiomyocytes of the adult heart prefer fatty acids as fuel to meet the high energy demand of mechanical work (99). The switch of fuel preference in fetal and adult cardiomyocytes is likely related to greater availability of oxygen in the adult heart, which is necessary for efficient burning of fatty acids. Reliance on anaerobic glycolysis, such as during extended periods of work, produces a condition of ATP deficit and excess lactic acid, which exacerbates cellular stress (100). Coincidentally, the fuel preference of cardiomyocytes in the failing adult heart shifts from fatty acids to glucose, reflecting reduced oxygen availability and oxidative capacity (99, 101). Amino acids generated by autophagy may be an important source of energy for cardiomyocytes during severe cellular stress conditions. In general, fuel selection in cardiomyocytes is influenced by nutrient availability, namely excess or deficiency of fatty acids, glucose, oxygen, and calcium. Long term consumption of the Western-style diet, a condition of excess energy input, is typically associated with the development of cardiac ischemia which often end in heart failure (1, 7, 102, 103), although the morbidity of acquired cardiac disorders is influenced by gene variations (104).

Cardiac energetics has been the topic of intensive studies for decades. Early studies employed intact hearts taken from animals to measure energy requirement and utilization (105). More recent studies using transgenic and targeted gene disruption technologies in mice permitted the direct evaluation of many genes on cardiac metabolic efficiency and disease susceptibility (106). Animal studies, in conjunction with studies using isolated cardiomyocytes, have revealed that cellular stress is a key feature that initiates a pathogenesis of acquired cardiac disorders (107). Even though cardiomyocytes exhibit a preference for fatty acids as a fuel substrate, exposure to excessive amounts of fatty acids induce lipotoxicity due to elevation in the synthesis of lipid signaling molecules such as diacylglycerol and ceramide, accumulation of ROS, and increased fat synthesis and accretion (108, 109). Moreover, acyl-CoAs have been shown to act on histone deacetylases, affecting the epigenome and thus the gene expression program (110, 111).

In recent years, there has been substantial progress toward our understanding of how cardiomyocytes respond to cellular stress (3, 72, 79, 112). However, other cardiac cell types also contribute key energy-requiring roles to deliver heart function, such as ion transport to coordinate electrical conductance and cell proliferation during cardiac remodeling to maximize mechanical capacity while minimizing the negative impacts of changes in cardiomyocyte morphology. At very high levels, ROS induce an inflammatory response involving macrophages and cardiac fibroblasts (113, 114). Increased lactic acid synthesis has been shown to stimulate vascular smooth muscle cells (115). The mechanisms involved in orchestrating the coordinated adaptations of different cardiac cell types is not well understood.

## Cardiac electrical conductance and stress coping responses

**Figure 4:**
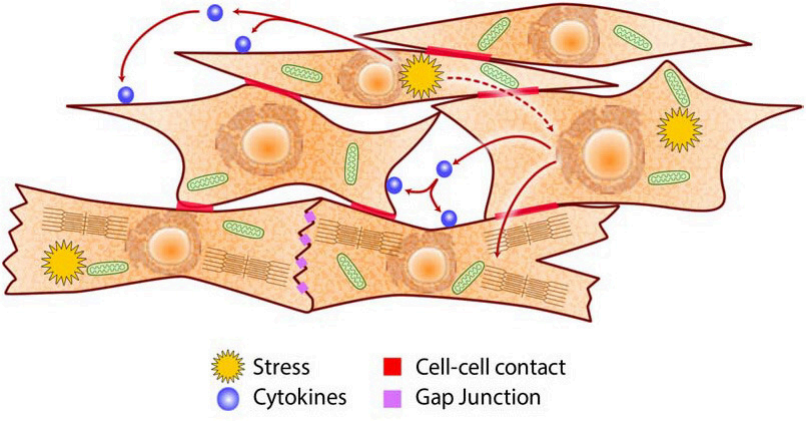
Schematic of intercellular communications in the heart. Cardiac cells communicate via cell-cell contacts (e.g., adhesion molecules, gap junctions), paracrine/autocrine signaling (e.g., cytokines, growth factors), and metabolites (e.g., ROS, adenosine, NO) to coordinate their specific roles in generating the heartbeat and in adapting to metabolic challenges.

Electrical impulses that drive the cardiac rhythm originate from SA node and then spreads rapidly to the pacemaker cells in the AV node. The pacemaker cells produce action potential that propagates to cardiomyocytes to initiate the influx of extracellular Ca^2+^ into the cardiomyocytes to drive the E-C coupling mechanism. The E-C coupling links the electrical activation of the surface membrane to mechanical force, i.e., the contraction. This process requires coordinated movement of Ca^2+^ at the level of cardiomyocyte CRN (Figure 2) (51). Gap junctions between cardiomyocytes are important for mediating electrical cell-cell communication by ion signal spread (Figure 4). These structures are not equally distributed across the myocardium but high numbers are found localized at the ends of cardiomyocytes. This arrangement can affect electrical and metabolic coupling of cardiac cells, and influence both the propagation of the conduction velocity and the direction of the signal spread along adjacent cardiomyocytes. Importantly, the function of gap junctions and expression of gap junction components are sensitive to cell stress (116, 117).

A growing list of potentially lethal cardiac arrhythmias including catecholaminergic polymorphic ventricular tachycardia, atrial fibrillation, long QT syndrome and hypertrophic cardiomyopathy, are linked to activation of cellular stress coping mechanisms triggered by environmental factors. Atrial fibrillation is one of the most common cardiac arrhythmias associated with stress and inflammation of cardiac tissue (118, 119). At the molecular level, dysregulation of Ca^2+^ handling in the SR is the major factor contributing to the development of atrial fibrillation (119, 120). Modification of Ca^2+^ handling proteins that lead to increased Ca^2+^ release from the SR result in the large rise in cytoplasmic and mitochondrial Ca^2+^ concentration (121–123) causing mitochondrial dysfunction, reduced ATP synthesis, increased ROS production (124–126), activation of UPR, attenuation of Ca^2+^-independent transient outward K^+^ channel and apoptosis (Figure 3). All these features have been associated with cardiac arrhythmias in failing hearts (122, 127, 128). Activation of UPR also drives acquired arrhythmias (122). For example, PERK activation downregulates the Na^+^ channels and decreases Na^+^ current density (127, 128), reduces cardiac K_v_4.3 channel conductance (128) and consequently cardiac conduction velocity. Inhibition of PERK prevents downregulation of these ion channels (128). Impaired glycosylation of the Na_v_1.5 contributes to stimulation of UPR due to misfolding and impaired trafficking of the ion channel also leading to arrhythmias (129).

Endothelial cells control vascular tone by releasing vasodilators including NO, prostacyclin and endothelin-1, angiotensin II which affect E-C coupling (130). Endothelial cells lack gap junctions and their communication with neighboring cells, in particular cardiomyocytes, must occur by gap junctions-independent mechanisms. Endothelin-1 secreted by endothelial cells mediates electrical remodeling during heart failure by downregulating the expression and phosphorylation of gap junction proteins connexin40 and connexin43 in cardiomyocytes, as well as by reducing Na_v_1.5 protein abundance and Na^+^ channel conductance (131). In contrast, cardiac fibroblasts are directly coupled to cardiomyocytes by gap junctions (132) and also express voltage sensitive and mechanosensitive ion channels (133, 134) that likely modulate cardiac electric activity. Finally, resident cardiac macrophages were recently found in the distal AV node. These cells are permanently attached to AV node pacemaker cells via connexin43-containing gap junctions and are essential for both normal and aberrant cardiac electrical conduction (37).

Even though a large body of information on the biology of cardiac cells has been accumulated, the nature of cardiac cell type-specific stress sensors and the outcomes of their stress coping response mechanisms that deal with electrical properties of the heart are not well studied.

## Cardiac mechanical work and stress coping responses

The main role of cardiomyocytes is to carry out mechanical function, but these cells also contribute to electrical conductance. Mechanical coupling between cardiomyocytes ensures that the outcome of action potential pacing synchronizes cardiomyocyte contraction to produce a coherent heartbeat (135). Considering an average of 80 heartbeats per minute, cardiomyocytes are well equipped to deal with repeated stretch-induced mechanical stresses. Prolonged mechanical force overload activates UPR, induces inflammatory mediators and causes ventricular hypertrophy (136). Long-term mechanical stretch-induced ER/SR stress may also affect other cardiac cell types, although their sensitivity to this stressor is not known. Disruptions in E-C coupling cause ER/SR stress and congestive heart failure (137). Increased abundance of calreticulin, a Ca^2+^ binding ER/SR resident chaperone, is associated with the failing human heart (127, 138). In mice, forced overexpression of calreticulin in cardiomyocytes increases cardiomyocytes ER/SR Ca^2+^ capacity and mechanical work potential, but also activates the IRE1α branch of UPR and eventually leads to cardiomyopathy (116, 139). Calreticulin overexpression also causes the reduction of gap junction protein abundance in the heart indicating a defect in cell-cell communication (116).

Heart failure is a typical result of extended periods of extreme mechanical activity (140). Remarkably, mechanical unloading with left ventricular assist devices improves Ca^2+^ handling and reduces UPR status in myocardial tissue of patients with advanced heart failure (141). The activation of the PERK branch of UPR in cardiomyocytes is associated with pressure overload-induced congestive heart failure (142) and affects multiple cardiac genes including increased expression and stability of Na^+^ voltage-gated channel α5 and K_v_4.3 channel (127). Other molecular components of the CRN are also involved. For example, tribbles homolog 3 (TRB3), a neuronal cell death-inducible putative protein kinase, is increased by ER/SR stress in cultured cardiomyocytes where it plays a role in stretch-induced cardiomyocyte apoptosis (143). E3 ubiquitin-protein ligase, HMG-CoA reductase degradation 1 (Hrd1) and ERAD are essential components of UPR in cardiomyocytes. Hrd1 is involved in preserving heart structure and function in a mouse model of cardiac hypertrophy (144). Nogo-C, a ubiquitously expressed ER protein is necessary for ischemia-induced cardiomyocyte apoptosis and cardiac dysfunction (145). Eva-1 homolog A (EVA1A), an ER-associated protein, improves cardiac function and inhibits cardiac hypertrophy and fibrosis by increasing autophagy (146). Stress coping response mechanisms induce changes within the CRN of cardiac cells and promotion of molecular constituents designed to preserve heart function (Figure 3).

Cardiomyocytes are the workhorse of the cardiac mechanical function and considerable information about cellular stress coping response mechanisms in these cells have been gained. However, the link between UPR and mechanical function of the heart and how the cardiomyocyte stress status is communicated to other cardiac cell types under unfavorable metabolic situations remains to be established.

## Cardiac tissue remodeling and stress coping responses

Cardiac remodeling is initially an adaptive response to hemodynamic stress and cardiac injury. In association with neurohormonal activation, cardiac remodeling can lead to cardiac diseases as a result of changes in gene expression program (e.g., re-expression of fetal genes) and cell morphology (volume, mass, shape). Cardiac tissue remodeling is stimulated by a variety of stressful conditions such as myocardial infarction, hypertension, myocarditis, idiopathic dilated cardiomyopathy or volume overload, and is associated with increased collagen synthesis induced by TGFβ, endothelin-1, angiotensin II and connective tissue growth factors culminating in cardiac fibrosis (147, 148). Communication among cardiomyocytes, fibroblasts and endothelial cells influences the biology of the cardiac ECM and is a key aspect of cardiac remodeling (147, 149–151). Endogenous pluripotent cardiac stem cells play an important part in cardiac remodeling and repair as they are capable of differentiating into cardiomyocytes, smooth muscle cells and endothelial cells (152–155).

Cardiac fibroblasts are central to the development of cardiac fibrosis but cardiomyocytes, endothelial cells and macrophages also contribute to the process. Increased abundance of TGFβ promote fibroblast proliferation and differentiation to myofibroblasts, activation of metalloproteinases in response to activation of β-adrenergic, angiotensin II and endothelin I receptors (147, 156, 157). Cardiac remodeling is also characterized by changes in cardiomyocytes contractile proteins and alterations in the handling of Ca^2+^ within the cardiomyocyte CRN (65, 147). For example, while increasing calreticulin abundance in the adult cardiomyocytes improves ER/SR Ca^2+^ capacity and delays SOCE, it also stimulates UPR (**Box 2**, Figure 3) which promotes the increase of cardiac TGFβ abundance that in turn induces increased collagen deposition and severe cardiac fibrosis (116, 139). Remarkably, pharmacological inhibition of UPR activation with tauroursodeoxycholic acid (TUDCA), a proteostasis promoter (158), inhibits the activation of IRE1α branch of UPR and averts the development of cardiac fibrosis (139, 159). Resveratrol, another proteostasis promoter (158), similarly reduces cardiac fibrosis in transverse aortic constriction induced heart failure (160, 161).

Endothelial cells, in pressure overload stressed myocardium, contribute to cardiac remodeling by regulating cardiomyocytes growth and modulating mechanical/electrical functions in a paracrine fashion (30, 130, 162, 163). Stress-induced endothelial cells impact on cardiomyocytes, driving cardiac hypertrophy (164–166). The inhibition of UPR with proteostasis promoter 4-phenylbutyrate prevents endothelial cell apoptosis, inflammation in the aorta, and development of the thoracic aortic aneurysm associated with degeneration of vascular smooth muscle cells (167). Endothelial-to-mesenchymal transition gives rise to cells that have fibroblast-like characteristics but still express endothelial markers, further enhancing cardiac remodeling and fibrotic events (168). Macrophages contribute to myocardial infarction-induced cardiac remodeling after recruitment to the ischemic myocardium to promote infarct repair and healing (169).

Stress-induced cardiac remodeling involves many cardiac cell types and is coordinated via gap junction-dependent cell-cell communication as well as both paracrine and autocrine signaling. However, each of the different cardiac cell types likely elaborate unique adaptive outcomes that have consequences not only on their own metabolic status but also that of their neighboring cells.

## Coordination of cardiac cell roles in the heart

The heart is a remarkably elegant and adaptable machine that can maintain its blood pumping action under a wide variety of metabolic conditions. To deliver a single heartbeat, an action potential generated by AV node pacemaker cells that is triggered by influx of Na^+^, causes membrane depolarization that opens L-type Ca^2+^ channels on the surface membrane and T-tubules of cardiomyocytes. The resulting entry of Ca^2+^ induces the opening of RyR Ca^2+^ channel to release Ca^2+^ into the cytoplasm, initiating muscle contraction. To relax, the Ca^2+^ is removed from cytoplasm by SERCA and Na^+^/Ca^2+^ exchanger. The Ca^2+^ signal is propagated as a wave via gap junctions along adjacent cardiomyocytes to drive contraction and relaxation cycles of the whole organ. For the heart to function properly, Ca^2+^ handling by cardiomyocytes must be tightly regulated, and Ca^2+^ concentration must be appropriately high in systole and low in diastole. Thus, the heartbeat is a result of cooperative roles carried out by several cell types that communicate with each other using a variety of methods (Figure 4). It is crucial to ensure a sufficient supply of ATP in the heart to meet changing metabolic demands, since a deficit in energy will cause the breakdown in the coordination of cardiac cell roles which eventually culminates in the loss of heart function and death of the organism.

The heart is dependent on key nutrients (minerals such as calcium, potassium, and magnesium for conduction of electrical impulse; simple sugars, fatty acids and amino acids as sources of energy to fuel muscle contraction and relaxation; iron and oxygen for efficient synthesis of ATP) to accomplish its function. Severe deficiency or large excess of nutrients, which originate from external sources, cause loss of nutrient/energy homeostasis that in turn cause the disruption of proteostasis and lipidostasis in the CRN (Figures 2, 3) (7, 170–172). How does the heart perform its function continuously when the cells that constitute the organ are faced with cellular stress? Cardiac cells experiencing stress not only act to restore their own cellular homeostasis but also emit signals that induce pathways in neighboring cells (Figure 4) designed to preserve the function and integrity of the organ as a whole. Autocrine/paracrine signaling involves cytokines such as angiotensin II, endothelin-1, TGFβ, VEGF, FGF, PDGF, MCP-1, and leptin that interact with cell surface receptors. Endothelial cells control cardiomyocytes E-C coupling by releasing vasodilators including NO, prostacyclin and endothelin-1, angiotensin II, natriuretic peptides, ROS, kinins, adenosine, and others. Cytokine activated endothelial cells express adhesion molecules (intracellular adhesion molecule-1, ICAM-1; vascular cell adhesion molecule-1, VCAM-1) that recruit and promote infiltration of immune cells into myocardium in response to stress stimuli (173). E-C coupling is sensitive to metabolic stress that induces signaling cascades that modify ion channels abundance and behavior, causing electrophysiological and structural remodeling in the heart that promote arrhythmogenesis (174, 175). Communication between cardiomyocytes, fibroblasts and endothelial cells, is a key aspect of cardiac remodeling and influences biology of the ECM components in the heart (Figure 4). Under stress conditions there is increased release of TGFβ from macrophages and fibroblasts that promotes fibroblasts activation, differentiation into myofibroblasts, production of ECM and cardiac remodeling. Upon cardiac injury there is an increased abundance of ECM promoting cardiac remodeling (e.g., fibrosis, angiogenesis) or ECM break down releasing cytokines bound to the ECM. Increases in NO release from endothelial cells activate guanylyl cyclase causing increased formation of cGMP and vasodilation increasing coronary blood flow to relieve ischemia. This ultimately results in promotion of protein synthesis including sarcomere proteins, hypertrophy, activation of the fetal cardiac gene expression program, fibrillogenesis and angiogenesis. Metabolites such as adenosine, NO, and ROS released by one stressed cardiac cell interact with transporters and receptors that activate signaling cascades and modify metabolic pathways of another cardiac cell. This unique arrangement of cardiac cells and specific signaling system (Figure 4) developed for cell-cell communication via direct contacts or via autocrine/paracrine signaling, are fundamental for the function of the heart as an organ and play important role under stress conditions in helping to maintain heart function.

It is likely that altered cellular function induced by stress coping response strategies (Figure 3) are meant to operate only during stress conditions. In the case of nutrient excess as in diet-induced cardiac disorders, cardiac cells are subject to prolonged exposure of nutrient overload causing persistence of altered metabolic functions that in the end drives the pathogenesis of cardiac disease (3, 7). In animal models of heart failure, administration of proteostasis promoters, such as TUDCA and resveratrol (158), have demonstrated efficacy in preventing the progression of cardiac fibrosis indicating that it is possible to delay terminal heart failure through therapeutic manipulation of stress coping response mechanisms such as UPR (139, 159–161). At present, it is not clear how the roles of the different cardiac cell types are modified by long-term cellular stress, or how these cells effectively communicate to coordinate their activities under unfavorable conditions.

## Challenges

Many animal models (mouse, rat, hamster, rabbit, dog, and pig) are commonly used in cardiovascular research (176, 177). Transgenic and targeted gene disrupted mice have permitted the evaluation of the function of virtually any gene *in vivo*, and together with primary and established cell cultures (cardiomyocytes, cardiac fibroblasts, vascular cells, and more recently iPS-derived cardiomyocytes) (15), these experimental models have been instrumental in understanding the pathophysiology of cardiac diseases. Nevertheless, despite the massive amount of information that has been gathered so far about the cardiovascular system, gaps in knowledge regarding cell-cell communication and how cardiac cell roles are coordinated still exist. One challenge that is particularly difficult to overcome relates to the inherent limitation of animal models, even humanized animal models, to fully recapitulate human biology. The impact of biological sex as an important modifier is another shortcoming of the existing knowledge base. Heart disease develops and presents differently in males and females (178–181). Yet little is known about the precise mechanisms that underlie sex differences and how these impact on prognosis, morbidity or therapeutic choices. There is evidence showing that male and female cardiac cells have distinct gene expression programs (182), and therefore differ in metabolic capacity. Fortunately, there is now a movement to include both sexes, not only in *in vivo* studies but also in *in vitro* studies, as well as to analyze their responses separately. The influence of extrinsic factors should also be taken into careful consideration. For example, palmitic acid has been the choice energy substrate used in numerous metabolic studies employing the isolated perfused heart model (183), yet palmitic acid is a potent inducer of UPR, autophagy, Ca^2+^ depletion, apoptosis and mitochondrial stress (184–190). Moreover, the importance of the extracellular matrix and intercellular communication among cardiac cells is not adequately addressed in many experimental designs. Cardiac cells grown in tissue culture lack important contextual information, such cell-cell contact between different cell types (Box 1, Figure 4) and other cues that indicate the metabolic status of neighboring cardiac cells as well as the heart itself. Analysis of abundance or phosphorylation status of specific proteins in cardiac tissue homogenates does not provide information about the status of specific mechanisms, such as stress coping responses (Figure 3), operating in each of the different cardiac cell types. These considerations are vital to the understanding of how the different cell activities are integrated to sustain a continuous rhythm.

## Summary

The heart is able to perform its blood pumping function due to the collaboration of different cell types that make up its structure. Specialized cell types are responsible for the principal activities of cardiac tissues, namely generation and propagation of the electrical impulse, facilitation of the rhythmic contraction and relaxation cycles, and the orchestration of the cardiac remodeling, which all operate coordinately to maintain a regular heartbeat throughout the lifetime of the organism. In response to stress inducers, especially to nutrient overload which disrupts cellular energy homeostasis, all cells employ the same basic cellular stress coping response mechanisms that operate in various organelles to sense and rectify metabolic disturbances in the cellular reticular network. In the context of the heart, the manifestations of stress coping strategies likely differ depending on the coping strategy outcomes of the different cardiac cell types, and thus may underlie the development of distinct cardiac disorders. Adaptive outcomes normally cease to operate when cellular stress is resolved, whereas long term exposure of cardiac cells to stress induce persistent changes in cell activity that eventually promote heart disease. In the case of nutrient excess, reduction in the intake of energy dense nutrients may be the first and crucial step toward the recovery of normal cardiac cell function. In addition, it may also be possible to delay complete heart failure in advanced cases of acquired cardiac diseases by simultaneous therapeutic targeting of cellular stress coping mechanisms operating in various organelles in order to accelerate the recovery of proteostasis and lipidostasis.

## Author contributions

All authors listed have made a substantial, direct and intellectual contribution to the work, and approved it for publication.

### Conflict of interest statement

The authors declare that the research was conducted in the absence of any commercial or financial relationships that could be construed as a potential conflict of interest.

## Abbreviations

ANF: Atrial natriuretic factor
AV: atrioventricular node
CHOP: C/EBP-homologous protein
CRN: cellular reticular network
ECM: extracellular matrix
E-C: excitation-contraction
ER: endoplasmic reticulum
ERAD: ER associated protein degradation
iPS: inducible pluripotent stem cells
IRE1α: inositol-requiring kinase 1α
MCS: membrane contact sites
MMPT: mitochondrial membrane permeability transition
ORAI: Ca^2+^ release-activated Ca^2+^ channel protein
PERK: ER kinase dsRNA-activated protein kinase-like ER kinase
ROS: reactive oxygen species
RyR: ryanodine receptor
SA: sinoatrial node
SR: sarcoplasmic reticulum
SERCA: ER/SR Ca^2+^-transport ATPase
SOCE: store-operated Ca^2+^ entry
STIM: stromal interaction molecule
TGFβ: transforming growth factor β
UPR: unfolded protein response.
